# Validation of a renal staging system and its association with renal amyloid deposition burden in AL amyloidosis

**DOI:** 10.1080/0886022X.2025.2499230

**Published:** 2025-05-19

**Authors:** Ying Yao, Shuang Wang, Dan-Yang Li, Xiao-Juan Yu, Jia-Yi Liu, Zhi-Xiang Qiu, Fu-De Zhou, Su-Xia Wang

**Affiliations:** ^a^ Laboratory of Electron Microscopy, Pathological Center, Peking University First Hospital, Beijing, P.R. China; ^b^ Renal Division, Department of Medicine, Peking University First Hospital; Renal Pathological Center, Institute of Nephrology, Peking University; Key Laboratory of Renal Diseases, Ministry of Health of China; Key Laboratory of CKD Prevention and Treatment, Ministry of Education of China, Beijing, P.R. China; ^c^ Department of Nephrology, Fuxing Hospital, the Eighth Clinical Medical College, Capital Medical University, Beijing, P.R. China; ^d^ Department of Hematology, Peking University First Hospital, Beijing, P.R. China

**Keywords:** Glomerular filtration rate, light chain amyloidosis, renal biopsy, staging system, survival analysis

## Abstract

**Objectives:**

This study evaluates the relationship between renal amyloid deposition burden in kidney biopsy and a renal staging system based on proteinuria and estimated glomerular filtration rate (eGFR) in AL amyloidosis.

**Methods:**

A total of 248 patients diagnosed *via* renal biopsy were included. The extent of amyloid deposition in glomeruli, blood vessels, and tubulointerstitium were evaluated semiquantitatively. The total amyloid load (TA) was defined by the sum of glomerular, vascular and interstitial deposits.

**Results:**

Patients were categorized into three renal stages: I, II, and III. Findings showed that scores of pathological parameters increased progressive with advancing renal stage. The median TA values were 6 (IQR 3–8) in Stage I, 7 (IQR 5–8) in Stage II, and 8 (IQR 7–11) in Stage III (*p* < 0.001). Baseline eGFR was inversely correlated with TA (*r* = −0.363, *p* < 0.001), while proteinuria showed no significant association. Cox regression analysis identified eGFR <50 mL/min/1.73 m^2^ as an independent risk factor for renal survival (HR, 6.519; 95% CI, 3.110–13.665; *p* < 0.001), whereas proteinuria did not show such an effect.

**Conclusions:**

These findings suggest that in the renal staging system, eGFR – but not proteinuria – is significantly associated with amyloid deposition and independently affects renal survival.

## Introduction

Immunoglobulin (Ig) light chain (AL) amyloidosis is defined by the accumulation of misfolded fibrillary proteins that originate from light chains or their fragments, produced by the clonal proliferation of plasma cells [1]. The kidneys are among the organs most commonly affected by AL amyloidosis, leading to varying levels of proteinuria and renal dysfunction [2]. Renal involvement significantly contributes to morbidity, and renal insufficiency limits treatment options [3]. Therefore, a staging or scoring system to predict renal outcomes is necessary.

Renal biopsy plays a crucial role in diagnosing amyloidosis, which is marked by amyloid deposits in all areas of renal tissue, including the glomeruli, blood vessels, and tubulointerstitium [4]. Prior research has shown a link between the extent of amyloid deposition in renal tissue and the resulting outcomes in renal amyloidosis [5–8]. Consequently, Rubinstein et al. proposed a new pathological scoring system to predict renal outcomes in AL amyloidosis. They found that the degree of amyloid deposition, indicated by an amyloid score (AS), was associated with the progression to end-stage kidney disease (ESKD), emphasizing the potential prognostic value of evaluating amyloid burden in renal biopsies [7].

A clinical renal staging system based on estimated glomerular filtration rate (eGFR) (≥50 or <50 mL/min/1.73 m^2^) and urine protein excretion (greater or less than 5 g/day) was initially proposed by Palladini et al. in 2014 [9]. This staging approach allows for the prediction of progression to dialysis in patients with AL amyloidosis at various renal stages and has been validated in European and Chinese cohorts [10–12]. However, it remains unclear whether this clinical renal staging system aligns with the amyloid deposition burden evaluated through pathological scoring in renal biopsies. Therefore, the aim of this study was to determine the relationship between this renal staging system and the extent of amyloid deposition evaluated from kidney biopsies in patients with AL amyloidosis.

## Materials and methods

### Population and study design

**Figure 1.**
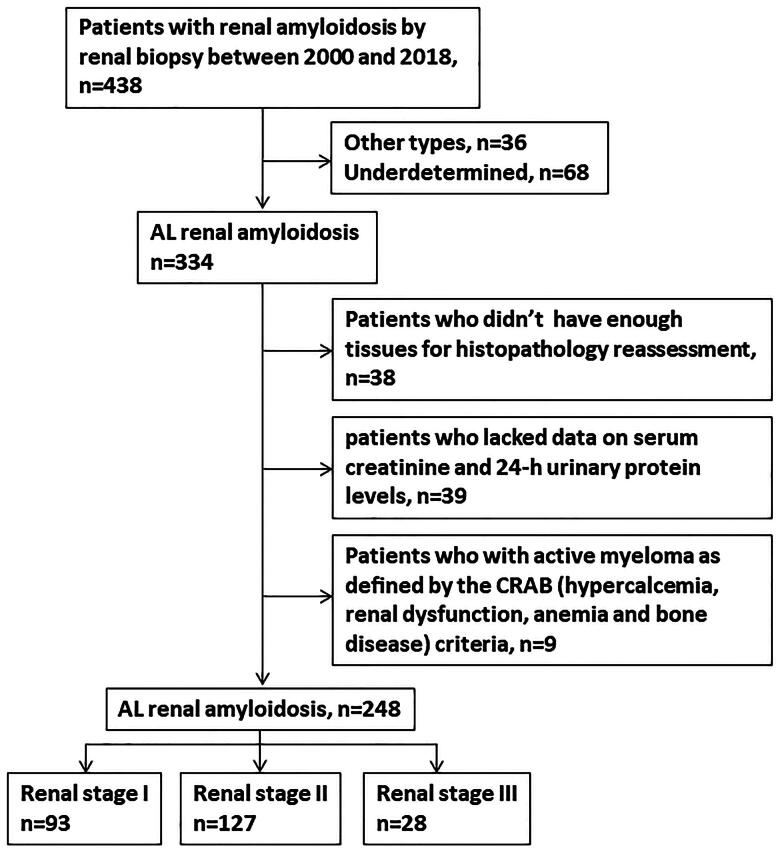
Flowchart for patient selection in the study.

A total of 438 patients diagnosed with renal amyloidosis and confirmed by renal biopsy at our center between January 1, 2000, and December 31, 2018, were assessed for inclusion in the study. Patients with non-AL amyloidosis or unclassified amyloidosis were excluded. Additionally, those lacking suitable tissue slides for histological reassessment, without data on 24-h urine protein levels and serum creatinine, or who met the CRAB criteria (hypercalcemia, impaired renal function, anemia, and bone disease) for active myeloma were also excluded. Ultimately, 248 patients were included in this retrospective cohort study. Details of the patient selection process are illustrated in Figure 1.

### Clinical and laboratory data

Clinical and laboratory data were retrospectively collected from the medical records. Sex, age, history of hypertension and diabetes mellitus, blood pressure, hemoglobin, serum creatinine, eGFR, serum albumin, serum alkaline phosphatase (AKP), cardiac troponins (cTnT), N-terminal pro-brain natriuretic peptide (NT-proBNP), serum-free light chain (FLC), immunofixation electrophoresis (IFE) of serum and urine, 24 h urine protein excretion, bone marrow aspiration and/or biopsy examination at the time of renal biopsy variables were used as baseline variables for analysis.

The eGFR was determined based on the Chronic Kidney Disease Epidemiology Collaboration equation. The definition of extrarenal organ involvement has been provided in previously conducted studies. Cardiac involvement was defined as a mean left ventricular wall thickness exceeding 12 mm in the absence of hypertension or other potential causes of left ventricular hypertrophy. Hepatic involvement was determined based on the presence of hepatomegaly evident on imaging studies (in the absence of heart failure) or a serum AKP level elevated to at least 1.5 times the upper limit of the institutional normal range [13,14].

Renal staging is defined by the criteria put forth in 2014, based on the presence of two risk variables, eGFR (<50 mL/min/1.73 m^2^) and proteinuria (>5 g/day), upon diagnosis; the renal stage can be classified as renal stage I (no risk variable), renal stage II (one variable), or renal stage III (both variables) [9].

### Diagnosis and typing of renal amyloidosis

Diagnosis of amyloidosis was made through renal biopsies showing positive Congo red staining with apple green birefringence under polarized light, further validated by the presence of non-branching fibrils measuring 8–12 nm in diameter on electron microscopy. Amyloid typing was conducted using immunofluorescence (IF) microscopy on frozen tissue or immunohistochemical (IHC) staining on paraffin sections. In cases where IF/IHC results were inconclusive (*n* = 8), immunoelectron microscopy was utilized. Additionally, laser microdissection and tandem mass spectrometry-based proteomics were employed for subtyping in some instances (*n* = 7).

### Scoring histopathological lesions and measuring renal amyloid deposition

The extent of glomerular amyloid (GA) deposition was scored on a scale from 0 to 4 based on the percentage of amyloid deposits relative to the total glomerular area, categorized as follows: 0 (absent), 1 (1%–10%), 2 (11%–25%), 3 (26%–50%), and 4 (more than 50%). Similarly, the degree of amyloid deposition in blood vessels (VA) was rated from 0 to 4 according to the percentage of amyloid deposits in the interlobular artery section area, using the same categories. The extent of interstitial amyloid (IA) deposition, inflammatory infiltration (Iinf), interstitial fibrosis, and tubular atrophy (Ifib) was also scored from 0 to 4 based on the percentage of lesion involvement [6,15]. The scores for GA, VA, and IA were summed to calculate the total amyloid load (TA) [6].

All histopathologic samples were reviewed and scored by a single renal pathologist who was unaware of the patients’ clinical information. To assess intra-rater reliability, 20 biopsies were reevaluated by the same pathologist. Additionally, a second renal pathologist independently reviewed a random selection of 20 biopsies to determine inter-rater reliability. Quantitation of renal amyloid deposition was conducted using computerized image analysis (Image-Pro Plus 6.0 software, Media Cybernetics, MD) on a random subset of 20 biopsies. The results from the computerized analysis were compared to those by the renal pathologist to assess inter-rater reliability.

### Treatment and outcome

The first-line treatment options included high-dose melphalan with autologous stem cell transplantation (HD-ASCT), chemotherapy without HD-ASCT (non-HD-ASCT), and supportive care only (no treatment) [16]. Reasons for not receiving treatment included death within 1 month of diagnosis due to advanced disease, multiorgan failure preventing treatment, and the patient’s preference. Various alternative therapies were employed in the non-HD-ASCT group, reflecting changing institutional policies over the study period. The selection criteria for HD-ASCT were based on the Mayo Clinic criteria [17]. The follow-up period was defined as the time from diagnosis to death or the most recent follow-up [18]. Renal survival was assessed based on the duration from diagnosis to the start of hemodialysis [9]. For the purpose of the renal survival analysis, patients who died without beginning dialysis were considered censored [19].

### Statistical analysis

Continuous variables are presented as mean ± standard deviation or median (interquartile range [IQR]), while categorical variables are shown as frequencies and percentages. The *t*-test, Mann–Whitney *U* test, and Pearson chi-square or Fisher’s exact test were utilized for comparisons between two groups. For comparisons among three different renal stages, one-way analysis of variance, Kruskal–Wallis test, and linear-by-linear association were applied. The correlation between variables was assessed using Spearman’s correlation coefficient. The Kaplan–Meier method was employed to generate overall survival and renal survival curves, with group differences analyzed using the two-tailed log-rank test. Cox models were used to identify baseline variables that predict renal survival and overall survival. The analyses were conducted with IBM SPSS Statistics version 25, and all hypothesis tests were considered significant at a threshold of 0.05.

## Results

### Comparing clinical characteristics across different renal stages

A total of 93 (36.5%), 127 (51.2%), and 28 (11.3%) patients were categorized into Stages I, II, and III according to the renal staging established by Palladini et al. [9]. The demographic and clinical characteristics of the patients are detailed in Table 1.

**Table 1.** Patient’s clinical characteristics.

Variable	All *n* = 248	Stage I *n* = 93	Stage II *n* = 127	Stage III *n* = 28	*p*-value
Male, *n* (%)	160 (64.5)	57 (61.3)	81 (63.8)	22 (78.6)	0.156
Age, years, mean ± SD	59.6 ± 10.1	59.9 ± 10.3	58.4 ± 9.6	63.3 ± 11.1	0.129
plasma cell, %, median (IQR)	3.0 (1.5–5.5)	3.5 (1.5–6.5)	2.5 (1–4.5)	2.5 (2–5.5)	0.268
Serum FLC involved, mg/L, median (IQR)	136 (56.35–247)	130 (63.4–347.6)	142 (36.2–247)	172 (105.3–306)	0.765
dFLC, mg/L, median (IQR)	101.7 (46–221.8)	105 (48.7–325.6)	102 (25.7–220.7)	72.6 (66.5–274.3)	0.983
Light-chain isotype (κ), *n* (%)	42 (16.9)	13 (14)^a^	19 (15)^a^	10 (35.7)	0.037
M protein type					
Free λ	47	15	29	3	
Free κ	9	3	5	1	
IgG λ	42	13	21	8	
IgA λ	21	8	11	2	
IgG κ	6	1	3	2	
IgM κ	1	0	1	0	
IgM λ	1	1	0	0	
IgG λ+ IgM λ	1	0	1	0	
SBP, mmHg, median (IQR)	117 (101.5–128)	110 (100–125)^a^	117 (100–124)^a^	128 (113–143)	0.001
DBP, mmHg, median (IQR)	73 (67–80)	70 (65–80)^a^	73 (67.5–80)^a^	80 (70–88)	0.022
UPE, g/day, median (IQR)	5.36 (3.5–7.6)	3.21 (2.0–4.2)^a,b^	6.81 (5.4–8.2)^a^	9.64 (7.4–12.7)	<0.001
UPE ≥5g, *n* (%)	137 (55.2)	0	109 (85.8)	28 (100.0)	
Serum creatine, mg/dL, median (IQR)	0.87 (0.7–1.2)	0.8 (0.6–1.0)^a^	0.9 (0.7–1.1)^a^	2.3 (1.6–3.4)	<0.001
eGFR, mL/min·1.73 m^2^, median (IQR)	89.3 (59.1–104.8)	91.3 (73.2–109.8)^a^	91.8 (64.9–104.6)^a^	28.4 (15.6–44.1)	<0.001
eGFR≤ 50mL/min·1.73 m^2^, *n* (%)	46 (18.5)	0 (0)	18 (14.2)	28 (100.0)	
Heart involvement, *n* (%)	71 (41.8)	26 (44.8)	33 (36.3)	12 (57.1)	0.727
Interventricular septal thickness, mm	1.14 ± 0.28	1.19 ± 0.29	1.08 ± 0.26	1.20 ± 0.28	0.163
Low voltage in limb leads, n (%)	51 (28.8)	21 (34.4)	20 (21.3)	10 (45.5)	0.038
NT-proBNP, ng/L, median (IQR)	51.1 (41.7–63.6)	49.7 (41.3–64.7)	50.9 (42–62.2)	55.3 (40.8–526.9)	0.794
Liver involvement, *n* (%)	50 (27.9)	16 (25.4)	26 (28.0)	8 (36.4)	0.378
Treatment, *n* (%)					0.059
0, no treatment	44 (25.6)	17 (29.3)	18 (19.6)	9 (40.9)	
1, non-HD-ASCT	102 (59.3)	33 (56.9)	56 (60.9)	13 (59.1)	
2, HD-ASCT	26 (15.1)	8 (13.8)	18 (19.6)	0 (0)	

SD: standard deviation; IQR: interquartile range; FLC: free light chain; dFLC: difference between involved (amyloidogenic) and uninvolved free light chain; SBP: systolic blood pressure; DBP: diastolic blood pressure; UPE: urinary total protein excretion; eGFR: estimated glomerular filtration rate; NT-proBNP: N-terminal pro-brain natriuretic peptide; non-HD-ASCT: chemotherapy treatment; HD-ASCT: high-dose melphalan with autologous stem cell transplantation.

^a^Compared with Stage III, *p* < 0.05; ^b^Compared with Stage II, *p* < 0.05.

Normal range for our laboratory: serum FLCλ, 5.71–26.3 mg/L; serum FLCκ, 3.3–19.4 mg/L; NT-proBNP, 0–125 ng/L.

In bone marrow aspirates, the median plasma cell count (*n* = 152) was recorded as 3.5 (IQR 1.5–6.5) for Stage I, 2.5 (IQR 1–4.5) for Stage II, and 2.5 (IQR 2–5.5) for Stage III, with a *p*-value of 0.268. Serum levels of involved free light chains (iFLC) (*n* = 34) and the dFLC (involved FLC minus uninvolved FLC; *n* = 34) were similar across the three stages, with *p*-values of 0.765 and 0.983, respectively. The proportion of patients with the κ light chain isotype was 14% in Stage I, 15% in Stage II, and 35.7% in Stage III, indicating a significant increase with advancing renal stage (*p* = 0.037) (Table 1).

The proteinuria levels (g/day) for Stages I, II, and III were 3.21 (IQR 2.0–4.2), 6.81 (IQR 5.4–8.2), and 9.64 (IQR 7.4–12.7), respectively, with *p* < 0.001 for all comparisons. Patients in Stage III had the highest serum creatinine levels (*p* < 0.001) and the lowest eGFR (*p* < 0.001), while renal function parameters were comparable between Stages I and II (Table 1).

Regarding extrarenal involvement, the rates of cardiac involvement (*n* = 170) were 44.8% in Stage I, 36.3% in Stage II, and 57.1% in Stage III, with a *p*-value of 0.727. The rates of hepatic involvement (*n* = 178) were 25.4% in Stage I, 28% in Stage II, and 36.4% in Stage III (*p* = 0.378) (Table 1).

Concerning first-line treatment received (*n* = 172), 26 patients, including 8 from Stage I, 18 from Stage II, and none from Stage III, underwent HD-ASCT. Furthermore, 102 patients, with 33 in Stage I, 56 in Stage II, and 13 in Stage III, received non-HD-ASCT regimens (including 31 on melphalan-based regimens, 46 on bortezomib-based regimens, 6 on thalidomide-based regimens, and 19 on other regimens). Additionally, 44 patients – 17 in Stage I, 18 in Stage II, and 9 in Stage III – received no treatment.

### Comparison of pathologic changes in renal biopsy across renal stages

The distribution of scores for GA, VA, IA, TA, Iinf, and Ifib among all patients is presented in Supplementary Table S1. The medians for GA, VA, IA, TA, Iinf, and Ifib were 3, 3, 1, 7, 1, and 1, respectively.

**Figure 2.**
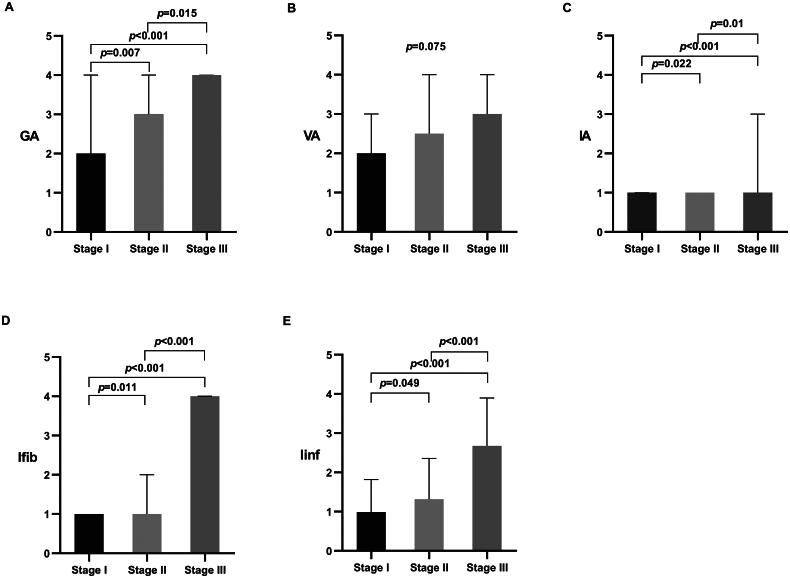
Comparison of pathological scores across different renal stages.

GA was 2 (IQR 1–4) in Stage I, 3 (IQR 2–4) in Stage II, and 4 (IQR 3.25–4) in Stage III, showing a significant difference between the groups (*p* < 0.001) (Figure 2(A)). The proportion of patients with GA ≥ 3 (the median of GA score) was 49.5% in Stage I, 70.1% in Stage II, and 92.9% in Stage III, indicating an increase with advancing renal stage (*p* < 0.001) (Table 2).

**Table 2.** Comparison of pathological parameters in kidney biopsy across the renal stages.

	Stage I *n* = 93	Stage II *n* = 127	Stage III *n* = 28	*p*-value
GA, score (IQR)	2 (1–4)	3 (2–4)	4 (3.25–4)	<0.001
GA ≥ 3, *n* (%)	46 (49.5)	89 (70.1)	26 (92.9)	<0.001
VA, score (IQR)	2 (1–3)	2.5 (1–4)	3 (2–4)	0.075
VA ≥ 3, *n* (%)	43 (46.2)	63 (49.6)	19 (69.2)	0.086
IA, score (IQR)	1 (0–1)	1 (1–1)	1 (1–3)	<0.001
IA ≥ 1, *n* (%)	67 (72.0)	112 (88.2)	28 (100)	<0.001
TA, score (IQR)	6 (3–8)	7 (5–8)	8 (7–11)	<0.001
TA ≥ 7, *n* (%)	38 (40.9)	70 (55.1)	23 (82.1)	<0.001
Ifib, score (IQR)	1 (1–1)	1 (1–2)	4 (2–4)	<0.001
Ifib > 1, *n* (%)	16 (17.2)	43 (33.9)	25 (89.3)	<0.001
Iinf, score (IQR)	1 (1–1)	1 (1–2)	2.5 (2–4)	<0.001
Iinf > 1, *n* (%)	12 (12.9)	38 (29.9)	22 (78.6)	<0.001

IQR: interquartile range; GA: the extent of glomerular amyloid deposition; VA: the extent of amyloid deposition in blood vessels; IA: the extent of interstitial amyloid deposition; TA: the total renal amyloid load; Ifib: the extent of interstitial fibrosis and tubular atrophy; Iinf: the extent of inflammatory infiltration.

For VA, scores were 2 (IQR 1–3) in Stage I, 2.5 (IQR 1–4) in Stage II, and 3 (IQR 2–4) in Stage III. Although there was a trend indicating an increase in VA with advancing renal stage, this was not statistically significant (*p* = 0.075) (Table 2, Figure 2(B)).

IA was 1 (IQR 0–1) in Stage I, 1 (IQR 1–1) in Stage II, and 1 (IQR 1–3) in Stage III, with a significant difference observed between the groups (*p* < 0.001) (Table 2, Figure 2(C)). The proportion of patients with IA ≥ 1 was 72%, 88.2%, and 100% in Stages I, II, and III, respectively, indicating an increase with advancing renal stage (*p* < 0.001) (Table 2). A similar trend was noted for Ifib and Iinf across the three groups, with both parameters reaching their highest levels in Stage III and lowest in Stage I, and the differences were statistically significant (Table 2, Figure 2(D and E)).

**Figure 3.**
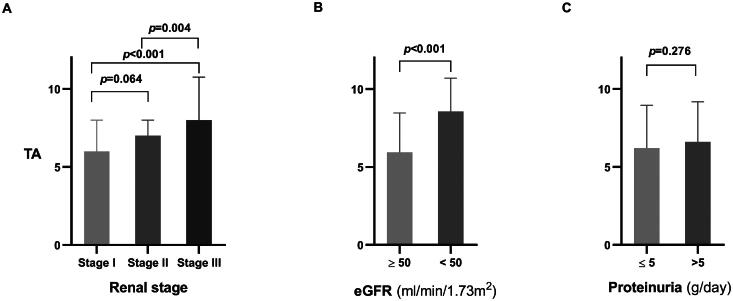
Comparison of TA among different renal stages (A), varying levels of eGFR (B), and different levels of proteinuria (C).

**Figure 4.**
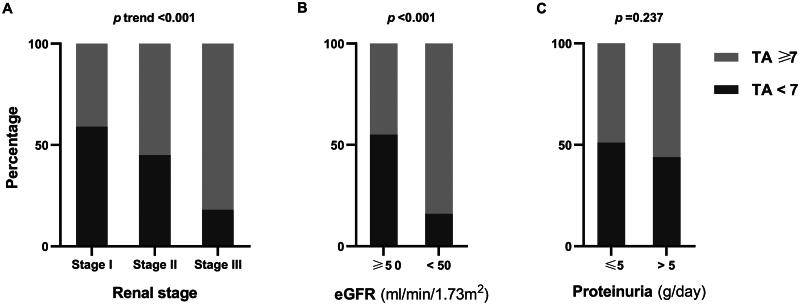
Comparison of the percentage of TA ≥7 across different renal stages (A), varying levels of eGFR (B), and different levels of proteinuria (C).

TA was significantly higher in Stage III (8 [IQR 7–11]) compared to Stage I (6 [IQR 3–8], *p* < 0.001) and Stage II (7 [IQR 5–8], *p* = 0.004). However, the difference in TA levels between Stages I and II was not significant (*p* = 0.064) (Table 2, Figure 3(A)). The proportion of patients with TA ≥ 7 (the median TA) was 40.9%, 55.1%, and 82.1% in Stages I, II, and III, respectively, showing a statistically significant trend of increasing TA with advancing renal stage (*p* < 0.001) (Table 2, Figure 4(A)).

### Association of proteinuria, eGFR, and amyloid deposition in renal biopsy

GA, VA, and IA were significantly higher in patients with eGFR <50 mL/min/1.73 m^2^ (Table 3). TA was 8 (IQR 7–10) in patients with eGFR <50 mL/min/1.73m^2^, which was significantly greater than the 6 (IQR 4–8) observed in patients with eGFR ≥50 mL/min/1.73m^2^ (*p* < 0.001) (Table 3, Figure 3(B)). The proportion of patients with TA ≥ 7 was significantly higher in those with eGFR <50 mL/min/1.73 m^2^ (84.8% vs.45.5%, *p* < 0.001) (Table 3, Figure 4(B)).eGFR showed a significant negative correlation with all pathological parameters, including GA, VA, IA, Iinf, and Ifib (*r* = −0.259, −0.283, −0.321, −0.558, and −0.537, all *p* < 0.001). There was also a significant negative correlation between eGFR and TA (*r* = −0.363, *p* < 0.001) (Table 4).

**Table 3.** Comparison of clinical and pathological parameters according to different levels of proteinuria and eGFR.

Variable	Proteinuria, g/day		*p*-value	eGFR, ml/min/1.73 m^2^		*p*-value
	≤5	>5		≥50	<50	
*n*	111	137		202	46	
Clinical parameter						
Age, years, mean ± SD	60.8 ± 10.1	58.6 ± 10.2	0.09	58.6 ± 9.9	64.0 ± 9.7	0.001
Male, *n* (%)	71 (64)	89 (65)	0.87	124 (61.4)	36 (78.3)	0.031
Proteinuria, g/day, median (IQR)	3.21 (1.97–4.13)	7.36 (6.0–10.0)	<0.001	5.26 (3.4–7.2)	6.42 (3.9–10.7)	0.029
eGFR, mL/min/1.73 m^2^, median (IQR)	85.6 (60.6–106.9)	89.7 (57.6–103.7)	0.826	93.6 (75.4–107.9)	29.4 (18.1–43.4)	<0.001
Heart involvement, *n* (%)	36 (48.0)	35 (36.8)	0.143	49 (37.1)	22 (57.9)	0.022
Liver involvement, *n* (%)	22 (27.5)	28 (28.3)	0.908	36 (25.7)	14 (35.9)	0.210
Treatment, *n* (%)			0.358			0.011
0, no treatment	20 (26.7)	24 (24.7)		32 (24.1)	12 (30.8)	
1, non-HD-ASCT	47 (62.7)	55 (56.7)		75 (56.4)	27 (69.2)	
2, HD-ASCT	8 (10.7)	18 (18.6)		26 (19.5)	0 (0)	
Pathological parameter						
GA, median (IQR)	3 (1–4)	4 (2–4)	0.003	3 (1–4)	4 (3–4)	<0.001
VA, median (IQR)	3 (2–4)	2 (1–3)	0.112	2 (1–3)	4 (3–4)	<0.001
IA, median (IQR)	1 (1–1)	1 (1–1)	0.025	1 (1–1)	1 (1–3)	<0.001
TA, median (IQR)	6 (4–8)	7 (5–8)	0.276	6 (4–8)	8 (7–10)	<0.001
TA ≥ 7, *n* (%)	54 (48.6)	77 (56.2)	0.236	92 (45.5)	39 (84.8)	<0.001
Ifib, median (IQR)	1 (1–2)	1 (1–2)	0.052	1 (1–1)	4 (2–4)	<0.001
Iinf, median (IQR)	1 (1–1)	1 (1–2)	0.220	1 (1–1)	3 (2–4)	<0.001

SD: standard deviation; IQR: interquartile range; eGFR: estimated glomerular filtration rate; HD-ASCT: high-dose melphalan with autologous stem cell transplantation; non-HD-ASCT: chemotherapy treatment; NA: indicates not applicable; GA: the extent of glomerular amyloid deposition; VA: the extent of amyloid deposition in blood vessels; IA: the extent of interstitial amyloid deposition; TA: total renal amyloid load; Ifib: extent of interstitial fibrosis and tubular atrophy; Iinf: extent of inflammatory infiltration.

**Table 4.** Correlation between proteinuria, eGFR, and pathological parameters in kidney biopsy.

Variable	Proteinuria, g/day		eGFR, ml/min/1.73 m^2^	
	*r*	*p*-value	*r*	*p*-value
GA	0.217	0.001	–0.259	<0.001
VA	–0.120	0.059	–0.283	<0.001
IA	0.166	0.009	–0.321	<0.001
TA	0.076	0.231	–0.363	<0.001
Ifib	0.129	0.042	–0.558	<0.001
Iinf	0.115	0.070	–0.537	<0.001

GA: the extent of glomerular amyloid deposition; VA: the extent of amyloid deposition in blood vessels; IA: the extent of interstitial amyloid deposition; TA: the total renal amyloid load; Ifib: the extent of interstitial fibrosis and tubular atrophy; Iinf: the extent of inflammatory infiltration.

GA and IA were higher in patients with proteinuria >5 g/day (*p* = 0.003, 0.025), while VA was lower in this group (*p* = 0.112) (Table 3). There was no significant difference in TA between patients with proteinuria >5 g/day and those with proteinuria ≤5 g/day (Table 3, Figure 3(C)). The proportion of patients with TA ≥ 7 was similar across different levels of proteinuria (56.2% vs 48.6%, *p* = 0.236) (Table 3, Figure 4(C)).

Spearman correlation analysis revealed a weak positive correlation between proteinuria and GA (*r* = 0.217, *p* = 0.001) and IA (*r* = 0.166, *p* = 0.009) and a weak negative correlation with VA (*r* = −0.120, *p* = 0.059). There was no correlation between proteinuria and TA (Table 4).

### Renal survival

**Figure 5.**
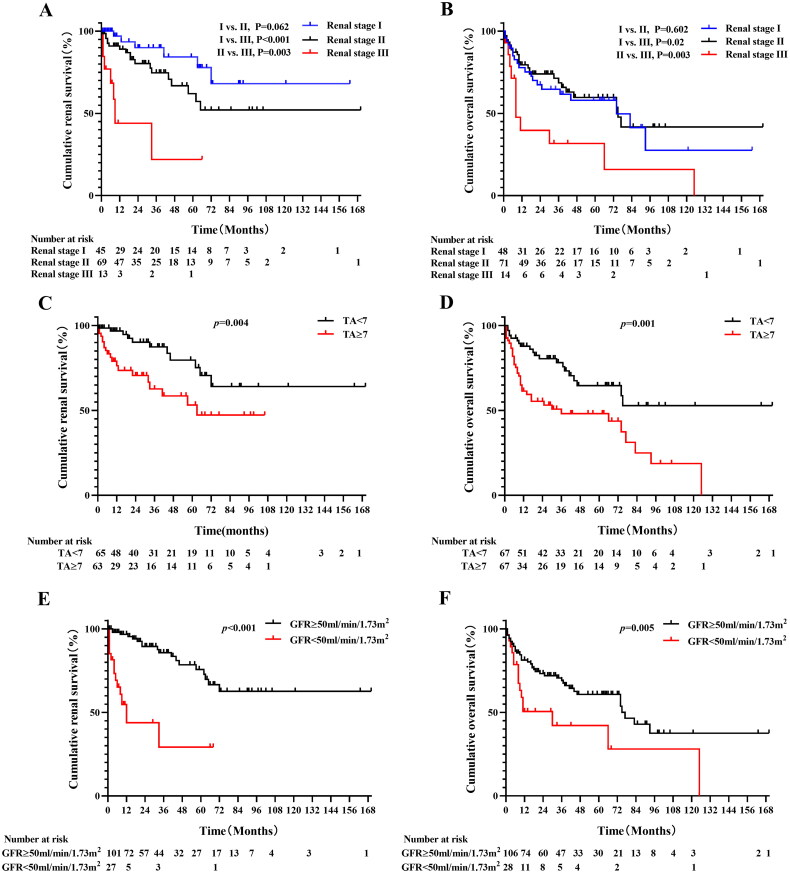
Renal survival and overall survival based on different renal stages (A) (B), varying levels of TA (C) (D), and different eGFR levels (E) (F).

The follow-up durations for Stages I, II, and III were 28 months (range 1–163), 21 months (range 1–170), and 9 months (range 1–125), respectively (*n* = 134). During the follow-up period, 32 patients progressed to dialysis, including 6 from Stage I, 19 from Stage II, and 7 from Stage III. Kaplan–Meier analysis revealed that the median time from diagnosis to the start of dialysis in Stage III was 9.1 months (95% CI: 5.8–12.5), which was significantly shorter than in Stage I (not reached [NR], *p* < 0.001) and Stage II (NR, *p* = 0.003) (Figure 5(A)).

Patients with TA ≥7 experienced worse renal survival compared to those with TA <7, with survival times of 62.8 months (75% CI: 11.8–NR) versus NR, *p* = 0.004 (Figure 5(C)). Additionally, patients with eGFR <50 mL/min/1.73 m^2^ had poorer renal survival, with survival times of 11.8 months (95% CI: 6.8–16.8) versus NR, *p* < 0.001 (Figure 5(E)). Renal survival rates were similar between patients with proteinuria >5 g/day and those with proteinuria ≤5 g/day, NR vs NR, *p* = 0.649 (Supplementary Fig. S1A).

Based on univariate Cox regression analysis, factors such as age, male sex, eGFR <50 mL/min/1.73 m^2^, heart involvement, Stage III, GA, IA, TA, Iinf, and Ifib were found to be associated with renal survival, while proteinuria and treatment were not. In the multivariate analysis, which included age, male sex, eGFR <50 mL/min/1.73m^2^, heart involvement, TA ≥ 7, Iinf >1, and Ifib >1, eGFR <50 mL/min/1.73m^2^ was identified as an independent risk factor for renal survival, with an HR of 6.519 (95% CI, 3.110–13.665), *p* < 0.001 (Table 5).

**Table 5.** Cox regression analysis of variables predicting renal survival.

Variable	HR	95%CI	*p*-value
Univariate analysis			
Age, year	1.046	1.007–1.086	0.017
Male sex	2.597	1.158–5.826	0.021
Proteinuria >5g/day	1.187	0.585–2.407	0.635
eGFR <50 mL/min/1.73 m^2^	6.930	3.316–14.482	<0.001
Renal stage			0.001
Renal stage I	references		
Renal stage II	2.292	0.913–5.754	0.077
Renal stage III	9.084	2.984–27.652	<0.001
Heart involved	2.454	1.160–5.191	0.019
Liver involved	1.405	0.602–3.280	0.432
Treatment, *n* (%)			0.267
0, no treatment	reference		
1, non-HD-ASCT	0.887	0.298–2.639	0.830
2, HD-ASCT	0.425	0.112–1.611	0.208
TA ≥7	2.862	1.374–5.960	0.005
Ifib >1	3.810	1.842–7.879	<0.001
Iinf >1	3.443	1.700–6.974	0.001
Multivariate analysis			
eGFR <50 ml/min/1.73 m^2^	6.519	3.110–13.665	<0.001

eGFR: estimated glomerular filtration rate; non-HD-ASCT: chemotherapy treatment; HD-ASCT: high-dose melphalan with autologous stem cell transplantation; TA: the total renal amyloid load; Ifib: the extent of interstitial fibrosis and tubular atrophy; Iinf: the extent of inflammatory infiltration; HR: Hazard Ratio.

### Overall survival

During the follow-up period, 56 patients died, including 20 in Stage I, 25 in Stage II, and 11 in Stage III (*n* = 134). The median overall survival for patients with Stage III was 8.4 months (95% CI, 3.8–13.1), significantly shorter than that for Stage I (74.3 months; 95% CI, 26.8–121.8; *p* = 0.02) and Stage II (74.5 months; 95% CI, 41.9–107; *p* = 0.003) (Figure 5(B)).

Patients with TA ≥ 7 had worse overall survival compared to those with TA < 7, with survival times of 36.4 months (95% CI, 0–93.5) versus NR (*p* = 0.001) (Figure 5(D)). Additionally, patients with eGFR <50 mL/min/1.73m^2^ had poorer overall survival, with 10.9 months (95% CI, 0–34.2) compared to 74.5 months (95% CI, 65.4–83.6) (*p* = 0.005) (Figure 5(F)). Overall survival rates were similar for patients with proteinuria >5 g/day and those with proteinuria ≤5 g/day, at 74.1 months (95% CI, 40.2–107.9) versus 74.3 months (95% CI, 27.8–120.8) (*p* = 0.884) (Supplement Figure S1B).

Univariate Cox regression analysis indicated that age, eGFR < 50 mL/min/1.73m^2^, Stage III, heart involvement, liver involvement, treatment, and TA ≥ 7 were factors related to overall survival. In the multivariate analysis, which included age, eGFR < 50 mL/min/1.73m^2^, heart involvement, liver involvement, treatment, and TA ≥ 7, treatment was found to independently affect overall survival (Table 6).

**Table 6.** Cox regression analysis of variables predicting overall survival.

Variable	HR	95% CI	*p*-value
Univariate analysis			
Age, year	1.031	1.002–1.061	0.036
Male sex	1.210	0.703–2.083	0.491
Proteinuria > 5 g/24 h	0.961	0.567–1.630	0.884
eGFR < 50 mL/min/1.73 m^2^	2.302	1.272–4.165	0.006
Renal stage			0.001
Renal stage I	reference		
Renal stage II	0.858	0.476–1.547	0.611
Renal stage III	2.477	1.178–5.208	0.017
Heart involved	3.033	1.735–5.304	<0.001
Liver involved	1.972	1.094–3.554	0.024
Treatment, *n* (%)			<0.001
0, no treatment	reference		
1, non-HD-ASCT	0.236	0.132–0.421	<0.001
2, HD-ASCT	0.058	0.019–0.176	<0.001
TA ≥7	2.543	1.468–4.403	0.001
Ifib >1	1.451	0.855–2.465	0.168
Iinf >1	1.427	0.825–2.466	0.203
Multivariate analysis			
Treatment, *n* (%)			<0.001
0, no treatment	reference		
1, non-HD-ASCT	0.235	0.129–0.428	<0.001
2, HD-ASCT	0.055	0.018–0.170	<0.001

eGFR: estimated glomerular filtration rate; non-HD-ASCT: chemotherapy treatment; HD-ASCT: high-dose melphalan with autologous stem cell transplantation; TA: the total renal amyloid load; Ifib: the extent of interstitial fibrosis and tubular atrophy; Iinf: the extent of inflammatory infiltration; HR: Hazard Ratio.

## Discussion

The Mayo Clinic 2012 staging system is the most commonly used method for predicting early mortality in patients with AL amyloidosis. Additionally, a renal staging system introduced by Palladini et al. [9] in 2014 has been established to estimate the risk of progression to dialysis within 2 years, as well as the annual risk, which has been highlighted in a recent review article on staging systems for AL amyloidosis published in the *New England Journal of Medicine* [20]. The renal outcome staging is based on two risk factors: proteinuria >5 g/day and eGFR <50 mL/min/1.73m^2^ at diagnosis. Patients are categorized into Stage I (no risk factors), Stage II (one risk factor), and Stage III (both risk factors). Higher stages indicate an increased likelihood of ESKD, a finding validated in multiple clinical studies, including a cohort of Chinese patients [10–12]. However, this staging system has yet to be validated in research focusing on the pathological features of renal biopsies. Renal biopsy is not only crucial for diagnosing amyloidosis but also holds prognostic significance.

In this study, it was confirmed that patients in Stage III experienced worse renal survival. An analysis of pathological features across different renal stages revealed that this staging system correlates with the extent of amyloid deposition, showing a trend of increasing TA with advancing renal stage.

In this renal staging system, we found that baseline eGFR at the time of renal biopsy was significantly related to the level of amyloid deposition, whereas proteinuria was not. Specifically, higher TA levels were associated with lower eGFR. Our results closely align with Hoelbeek’s study, which demonstrated that the AS, which encompasses mesangial, capillary, interstitial, vascular, and other types of amyloid involvement, correlated with eGFR but not with proteinuria at diagnosis [8]. The link between eGFR and amyloid deposition may be explained by the hypothesis that amyloid deposits disrupt tissue architecture, leading to organ dysfunction [21].

Survival analysis in our study showed that baseline eGFR was a critical factor affecting renal survival, while proteinuria was not significant. Patients with eGFR <50 mL/min/1.73m^2^ had poorer renal survival, even after adjusting for treatment, heart involvement, and age. This finding is consistent with Hoelbeek’s study, which also found a connection between AS and renal outcomes [8]. Additionally, our study noted that patients with Stage III disease exhibited worse renal and overall survival, likely due to their poorer renal function, as all Stage III patients had eGFR <50 mL/min/1.73 m^2^.

Palladini et al. found that proteinuria independently predicted the progression to dialysis in AL amyloidosis, with a thresholds of 5 g/24 h for urinary protein loss as the best discriminator for identifying patients who progressed [9]. In contrast, our study did not find a relationship between proteinuria and renal outcomes. These discrepancies may be attributable to differences in the study populations. The Palladini scoring system has been established in patients with AL amyloidosis cohorts receiving treatment. On the other hand, our cohort included a substantial proportion of patients who did not receive any treatment (44/172; 26%). Moreover, proteinuria did not correlate with TA in renal tissue in our study. Similar to the findings in studies by Hoelbeek’s and Wu’s [8,22], there was no evidence of a correlation between proteinuria and the AS or the composite scarring injury score as defined by Rubinstein et al. [7] in patients with AL amyloidosis.

Renal biopsy is crucial for diagnosing amyloidosis, as the extent of amyloid deposition in renal tissue significantly correlates with renal damage and outcomes [5–7,23–25]. This study confirmed that the TA, which includes amyloid deposition in the glomeruli, vasculature, and interstitium, is linked to the progression to ESKD. Kaplan–Meier analysis indicated that a higher TA, above the median, associated with increased amyloid deposition and more advanced stages, was correlated with poorer renal and overall survival. Furthermore, our study also found a significant relationship between Ifib and baseline eGFR. Previews research has suggested that tubulointerstitial fibrosis is strongly associated with the risk of ESKD across various diseases and represents a late event in kidney amyloidosis [7,26–28].

This study shows that baseline eGFR is significantly associated with the level of amyloid deposition in renal biopsy and is a risk factor affecting renal survival. This indicates that eGFR serves as a valuable clinical indicator for a worse prognosis and is an important reference for managing patients who have not undergone renal biopsy.

However, the study has two limitations. First, only 34 out of 248 patients had their FLC results available. Second, treatment responses were not documented. Since these factors are critical predictors of prognosis for AL amyloidosis, these limitations could impact the evaluation of prognosis.

In conclusion, a trend of increasing TA progression in the renal stages was noted. Among the two criteria in the staging system, only eGFR was identified as the key factor significantly related to the level of amyloid deposition and independently affecting renal survival.

## Supplementary Material

Supplemental Material

## Data Availability

The datasets analyzed during the current study are available from the corresponding author on reasonable request.
