# Supplementary material for: Validation of a renal staging system and its association with renal amyloid deposition burden in AL amyloidosis
Source: Ren Fail. 2025 May 19;47(1):2499230. doi: 10.1080/0886022X.2025.2499230 (PMC12090301; doi:10.1080/0886022X.2025.2499230)
Supplement: Supplemental Material [file IRNF_A_2499230_SM1747.doc]

**Supplement Table S1.** Pathological parameters in kidney biopsy for all patients

|  | Median (IQR) | Distribution of each score, n (%) | | | | |
| --- | --- | --- | --- | --- | --- | --- |
|  |  | 0 | 1 | 2 | 3 | 4 |
| GA | 3 (2-4) | 5 (2.0) | 48 (19.4) | 34 (13.7) | 50 (20.2) | 111 (44.8) |
| VA | 3 (1-4) | 19 (7.7) | 47 (19.0) | 57 (23.0) | 60 (24.2) | 65 (26.2) |
| IA | 1 (1-1) | 41 (16.5) | 171 (69.0) | 13 (5.3) | 6 (2.4) | 17 (6.9) |
| Ifib | 1 (1-2) | 12 (4.8) | 152 (61.3) | 31 (12.5) | 15 (6.0) | 38 (15.3) |
| Iinf | 1 (1-2) | 43 (17.3) | 133 (53.6) | 37 (14.9) | 13 (5.2) | 22 (8.9) |
| TA | 7 (5-8) | - | - | - | - | - |

Abbreviations: GA, the extent of glomerular amyloid deposition; VA, the extent of amyloid deposition in blood vessels; IA, The extent of interstitial amyloid deposition; Ifib, the extent of interstitial fibrosis and tubular atrophy; Iinf, the extent of inflammatory infiltration; TA, the total renal amyloid load; IQR, interquartile range.
